# Resistance related metabolic pathways for drug target identification in *Mycobacterium tuberculosis*

**DOI:** 10.1186/s12859-016-0898-8

**Published:** 2016-02-08

**Authors:** Ruben Cloete, Ekow Oppon, Edwin Murungi, Wolf-Dieter Schubert, Alan Christoffels

**Affiliations:** South African Medical Research Council Bioinformatics Unit, South African National Bioinformatics Institute, University of the Western Cape, Bellville, South Africa; Department of Biotechnology, University of the Western Cape, Bellville, South Africa; Current address: Department of Biochemistry, University of Pretoria, Pretoria, South Africa; Current address: Department of Biochemistry, Egerton University, Njoro, Kenya

## Abstract

**Background:**

Increasing resistance to anti-tuberculosis drugs has driven the need for developing new drugs. Resources such as the tropical disease research (TDR) target database and AssessDrugTarget can help to prioritize putative drug targets. Hower, these resources do not necessarily map to metabolic pathways and the targets are not involved in dormancy. In this study, we specifically identify drug resistance pathways to allow known drug resistant mutations in one target to be offset by inhibiting another enzyme of the same metabolic pathway. One of the putative targets, Rv1712, was analysed by modelling its three dimensional structure and docking potential inhibitors.

**Results:**

We mapped 18 TB drug resistance gene products to 15 metabolic pathways critical for mycobacterial growth and latent TB by screening publicly available microarray data. Nine putative targets, Rv1712, Rv2984, Rv2194, Rv1311, Rv1305, Rv2195, Rv1622c, Rv1456c and Rv2421c, were found to be essential, to lack a close human homolog, and to share >67 % sequence identity and >87 % query coverage with mycobacterial orthologs. A structural model was generated for Rv1712, subjected to molecular dynamic simulation, and identified 10 compounds with affinities better than that for the ligand cytidine-5′-monophosphate (C5P). Each compound formed more interactions with the protein than C5P.

**Conclusions:**

We focused on metabolic pathways associated with bacterial drug resistance and proteins unique to pathogenic bacteria to identify novel putative drug targets. The ten compounds identified in this study should be considered for experimental studies to validate their potential as inhibitors of Rv1712.

**Electronic supplementary material:**

The online version of this article (doi:10.1186/s12859-016-0898-8) contains supplementary material, which is available to authorized users.

## Background

*Mycobacterium tuberculosis*, the causative agent of tuberculosis (TB), is responsible for around two million deaths and nine million new cases annually. South Africa (SA) is one of the worst affected TB countries [1] and was an epicentre for an HIV-associated, extensively drug-resistant TB (XDR-TB) outbreak in 2005 within the KwaZulu Natal (KZN) province [2]. Three *M.tuberculosis* strains were subsequently isolated from sputum of HIV co-infected patients from KZN. These strains represent three levels of varying drug resistance phenotypes namely; susceptible, multiple drug resistant (MDR) and XDR TB [3].

Generally, TB strains are classified as MDR if they are resistant to first-line drugs Isonaizid (INH) and Rifampicin (RIF), and as XDR if they are additionally resistant to one of the second-line injec drugs Capreomycin, Kanamycin or Amikacin and at least one fluoroquinolone drug [1]. Increasing resistance to anti-TB drugs means that the need for novel drugs is growing in urgency.

**Fig. 1:**
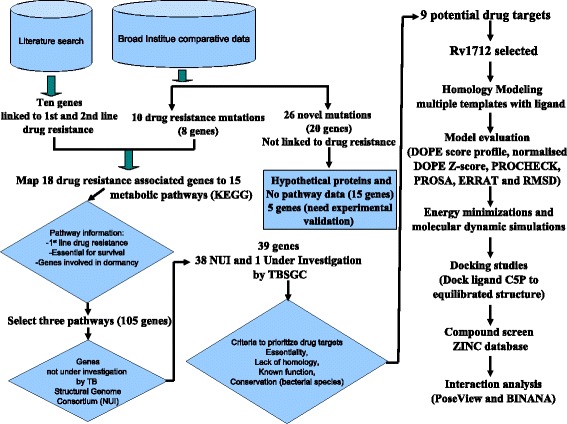
A workflow illustrating the different steps taken to identify potential drug targets for *M. tuberculosis,* homology modeling, molecular dynamics, docking strategy and interaction analysis for one target Rv1712

Current anti-TB drugs target information-processing DNA and RNA polymerase or DNA gyrase [4]. Drugs could, however, alternatively target metabolic pathways unique to this pathogen by comparing host and pathogen metabolism [5, 6]. The tropical disease research (TDR) target database and AssessDrugTarget can help in prioritizing putative drug targets by assigning a set of weighted criteria [7, 8], though most of these targets do not map to metabolic pathways and are not involved in dormancy. In this study, we specifically identify drug resistance pathways to allow known drug resistant mutations in one target to be offset by inhibiting another enzyme of the same metabolic pathway. Putative targets were filtered to exclude non-viable candidates based on essentiality for survival, lack of homology to human host, known biological function and conserved between mycobacterial species (Fig. 1). One of the proposed targets, Rv1712 was analyzed by Caceres et al. [9]. However, the authors did not deposit their homology models. Furthermore, their MD simulations were insufficient at 3 ns. The published homology modeling data for Rv1712 in the absence of any experimental data was insufficient as a starting point for identifying potential inhibitors. In this study, Rv1712 was screened for potential inhibitors using a strategy that included molecular modeling, molecular dynamics and *in silico* docking of potential inhibitors.

## Results

### Genome comparisons and pathway analysis

Genomic data from different TB strains previously confirmed ten point mutations in eight genes involved in first and second-line drug resistance and computationally identified 26 novel mutations in 20 genes in MDR and XDR KZN strains (https://www.broadinstitute.org). Further literature searches identified ten genes linked to resistance to first and second-line anti-TB drugs [10]. In this study, we exclusively focussed on the 18 experimentally verified drug resistance genes. Using the Kyoto Encyclopedia of Genes and Genomes (KEGG) database [11], twelve of the resulting 18 gene products linked to drug-resistance were mapped to 15 metabolic pathways, while six could not be assigned to a KEGG pathway (Additional file 1: Table S1). Three of the pathways, pyrimidine (42 genes), oxidative phosphorylation (47 genes), and nicotinate and nicotinamide metabolism (16 genes) were selected for their functional importance to bacterial growth and the latent state [12–15] as well as their promise as targets in slow growing bacteria [12–15].

### Selection and prioritization of candidate genes

Of 105 gene products in the three selected pathways, 14 are known TB drug targets and were excluded from further analyses [10]. The remaining 91 genes were checked to avoid duplication of research efforts with the TB Structural Genome Consortium (TBSGC) [16] leaving 38 putative targets. Additionally, no crystal structure was available for the TBSGC-target Rv2984 and was therefore included in our analysis (Additional file 2: Table S2). Of these 39 targets, 17 are essential for *M. tuberculosis* survival based on a Rv number query search within the Tuberculist Web server, have known biological function and have no experimental 3D structure (Additional file 2: Table S2).

Of these, nine are not homologous to any human proteins (*p* < 0.0001). They share >67 % sequence identity identity and >82 % sequence coverage with orthologs from other mycobacterial species (Additional file 3: Table S3). Publicly available microarray data indicates that four of the nine genes, Rv1712, Rv2984, Rv1622c and Rv2421c, are up-regulated during dormancy (starvation, hypoxia and oleic acid, *p*-values between 0.02 and 10^−9^) while five, Rv2194, Rv1305, Rv1456c, Rv2195 and Rv1311, are weakly down-regulated (*p* < 0.01). Other analyses used to validate the selected targets included BLASTp searches against three species of human gut flora bacteria (*Staphylococcus aereus*, *Enterococcus faecalis* and *Escherichia coli)* and mouse proteins (*Mus musculus*), revealing no homology to any mouse proteins (Additional file 4: Table S4). However, six proteins showed varying degrees of sequence identity and coverage to some intestinal bacteria. Of the nine proteins, Rv1712, Rv1311 and Rv2421c share ~40 % sequence identity and 90 % sequence coverage to orthologs in all three intestinal bacteria, while Rv2984 and Rv1622c share 30–40 % sequence identity to orthologs in two bacteria, and Rv1305 is 55 % identical to an ortholog in host gut bacteria with 87 % sequence coverage (Additional file 4: Table S4). The three remaining proteins, Rv2194, Rv2195 and Rv1456c are without homologs in the three human gut bacteria (Additional file 4: Table S4). KEGG pathway maps for the nine gene products were generated using the *M. tuberculosis H37Rv* strain database (Additional file 5: Figure S1, Additional file 6: Figure S2 and Additional file 7: Figure S3). Interestingly, Rv1622c and Rv1456c are at the interface of two metabolic pathways (Additional file 8: Table S5).

### Template selection and model building

A search of the protein data bank (PDB) revealed 1Q3T, 2H92, 1CKE and 1KDO as homologs for Rv1712. An alignment of the five proteins indicated sequence identities of 43 % (1Q3T), 40 % (2H92), 39 % (1CKE) and 40 % (1KDO) making them useful modeling templates. Incorporating structural data reveals a P-loop (residues 10–16) in the N-terminal ATP-binding domain and residues in a nucleoside monophosphate (NMP) binding site to be highly conserved. The conserved active site residues shared by Rv1712 and its four templates potentially allows these residues to be used in identifying novel inhibitors for enzymes of Rv1712 in docking studies.

**Fig. 2:**
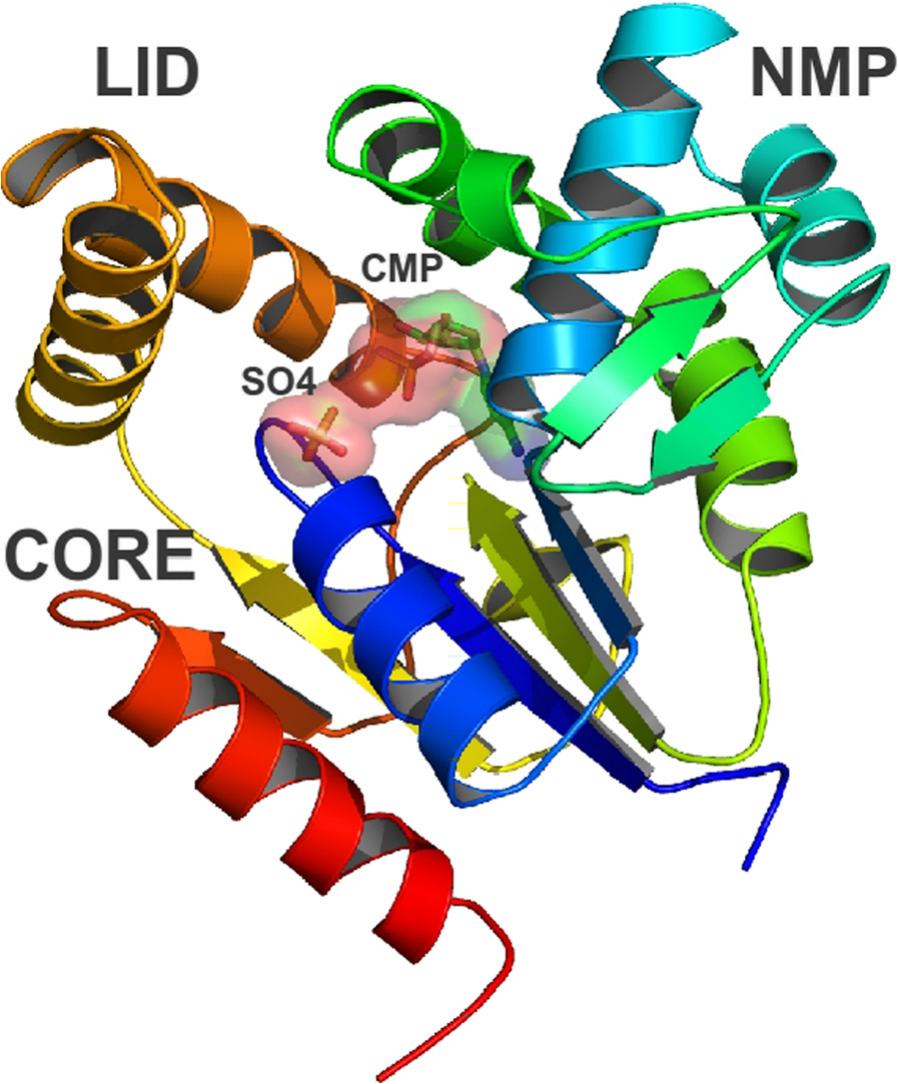
Ribbon diagram of the structural model of *M. tuberculosis* cytidilate monokinase (Rv1712). The CORE domain, NMP-binding domain and the LID domain are labelled as well as ligands CMP and SO4 shown as sticks. Figure generated using PyMOL [45]

Fifty structural models were constructed for Rv1712 and the model with the lowest discrete optimised protein energy (DOPE) score was selected for further analysis. The fold assessment score GA341 for the lowest DOPE model was equal to 1.0 suggesting that the correct fold was assigned to the protein Rv1712. The Rv1712 structural model contains nine α-helices and eight β-strands and encompasses a CORE (residues 10–16), an NMP-binding (33–100) and a LID (155–168) domain (Fig. 2). The NMP-binding domain is free to rotate during substrate binding and presumably recognizes both CMP or dCMP.

### Model quality validation

The DOPE score profile of the Rv1712 model is similar to that of templates 1Q3T, 1CKE, 1KDO and 2H92 (Additional file 9: Figure S4). Without high energy regions, the model would appear to be native like. The normalised DOPE Z-score was −0.77 indicating high model reliability. The model also conforms to permitted stereochemical restraints with 91.6 % of residues in most favoured and none in disallowed regions of the Ramachandran plot [17]. The quality factor calculated using the protein structure verification algorithm implemented in ERRAT [18] indicates that 85 % of residues in the 3D model have a low error rate compared to crystal structures of the same size and length. A Prosa Z-score for the Rv1712 model of −7.67 is comparable to those of the templates (−7.31 to −7.85). The root mean square deviation (RMSD) values obtained after aligning all atoms suggest that the Rv1712 model is most similar to 2H92 (0.53 Å) and 2O8R (0.62 Å) than to 1Q3T (3.25 Å) and 1CKE (2.33 Å). This indicates very little deviation from the main chain carbon atoms between target and template(s) suggesting homology and similarity between the structures. The inclusion of the recently solved crystal structure of cytidylate kinase from *M. smegmatis* did not significantly alter the Rv1712 model (Additional file 10: Figure S5). The Rv1712 model thus suitably approximates the actual protein structure (Fig. 2).

### Molecular dynamics

Trajectory analysis of the Rv1712 model in complex with ligand cytidine-5′-monophosphate (C5P) results in a rapid increase in RMSD during the first 2500 ps followed by a gradual decrease after 5000 ps for both the protein backbone atoms and ligand C5P carbon atoms (Additional file 11: Figure S6). The ligand RMSD was measured by performing a least squared fit to the starting conformation while allowing translation and rotation of all bonds within the ligand. An equilibrium phase was reached within 5000 ps suggesting that 30 ns was sufficient for stabilizing the structure. The average total energy and the potential energy reaches convergence at −1.1 × 10^6^ and −1.4 × 10^6^ KJ/Mole, respectively (Additional file 12: Figure S7). The RMS fluctuation for the C-alpha residues ranged between 0.07 and 0.38 nm and the radius of gyration for the molecule stabilized after 5000 ps fluctuating between 1.75 and 1.8 nm (Additional file 13: Figure S8 and Additional file 14: Figure S9). The simulation was repeated at random seed allowing all atoms to reach 300 K temperature and no significant drift was observed between the two trajectories.

### Virtual compound screening and interaction analysis

**Fig. 3:**
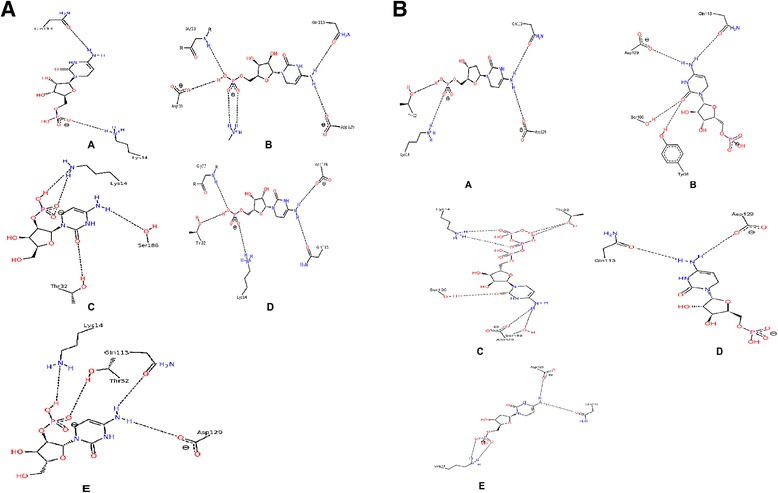
**a** Interactions between the top ranked compounds and active site residues of Rv1712. Panel *a* shows that the compound 03869482 is displaying two hydrogen bond interactions. Panel *b* shows that the compound 09007749 is displaying six hydrogen bond interactions. Panel *c* shows that the compound 04536469 is displaying four hydrogen bond interactions. Panel *d* shows that the compound 01785780 is displaying five hydrogen bond interactions. Panel *e* shows that the compound 04096023 is displaying four hydrogen bond interactions. The *dashed lines* represents hydrogen bonds. Figures generated using PoseView [50]. **b** Interactions between the top ranked compounds and active site residues of Rv1712. Panel *a* shows that the compound 01532581 is displaying two hydrogen bond interactions. Panel *b* shows that the compound 03861744 is displaying six hydrogen bond interactions. Panel *c* shows that the compound 13431062 is displaying four hydrogen bond interactions. Panel *d* shows that the compound 08952080 is displaying five hydrogen bond interactions. Panel *e* shows that the compound 03869816 is displaying four hydrogen bond interactions. The *dashed lines* represents hydrogen bonds. Figures generated using PoseView [50]

The lowest energy conformation obtained for docking C5P to the equilibrated structure was −7.3 kcal/mol (Additional file 15: Table S6). Screening 48 compounds from the ZINC database against the equilibrated structure yielded ten compounds with higher binding affinity values than −7.3 kcal/mol (Additional file 15: Table S6). Interaction analysis using PoseView shows that the natural substrate C5P forms one hydrogen bond with glutamine113 and a п-stacking interaction with tyrosine36. Salt bridge interaction analysis confirmed one positively charged residue arginine37 of Rv1712 with negatively charged C5P phosphate group. Compounds 03869482, 09007749, 04536469, 01785780, 04096023, 01532581, 03861744, 13431062, 08952080 and 03869816 respectively form 2, 6, 4, 5, 4, 4, 4, 7, 2 and 4 hydrogen bonds to Rv1712 (Fig. 3A and B). BINANA interaction analysis indicates a salt bridge between each compound and positively charged residues of Rv1712 such as lysine14, arginine37, aspartate129 and arginine185 (Additional file 15: Table S6). All compounds similarly retain a п-stacking interaction with tyrosine36 of Rv1712 (Additional file 15: Table S6).

## Discussion

This study focuses on metabolic pathways previously targeted by anti-TB drugs to increase the likelihood of selecting effective targets. Target validation included five filtering steps and two quality tests. Previous studies used AssessDrugTarget and the TDR target database to identify potential drug targets in pathogenic bacteria. This led to the transcription factor DevR and the enzymes GlnE and FbpD to be proposed as potential drug targets [7] based on their high ranking on the metabolic list despite neither being part of a metabolic pathway. The target protein FbpD was, moreover, listed among other essential enzymes in the *M. tuberculosis* species-specific list although not being an essential enzyme. Similarly, the biological role of the enzymes GlnE and FbpD remain unknown, limiting their use for biochemical and biophysical assays [19]. Our approach improves on these methods by selecting essential enzymes which have been functionally characterised thereby increasing the likelihood of selecting effective putative target candidates.

### Predicted sub-cellular localization of putative targets

Of the identified putative targets, six (Rv2984, Rv2194, Rv1305, Rv1622c, Rv2195, Rv1456c) are cytoplasmically membrane-associated proteins whereas three, Rv1712, Rv1311 and Rv2421c, are *bone fide* soluble, cytoplasmic proteins [20]. Membrane proteins are preferred drug targets as they are accessible from their extracellular surface without the drug having to be taken up or modified [21]. Unfortunately, membrane proteins are far more difficult to analyse structurally than soluble proteins hindering the assessment of their druggability [22]. Drug delivery strategies to intracellular targets have improved over the years and include the use of cell penetrating peptides, pH responsive carriers and endosome-disrupting agents [21].

### Cytidylate kinase and polyphosphate kinase as drug targets

Of the nine putative targets obtained using our approach, six (Rv1712, Rv2984, Rv2194, Rv1311, Rv1305 and Rv1622c) were previously identified as potential drug targets [4], underlining the robustness of our method. Of these, only Rv1712, cytidylate kinase, and Rv2984, polyphosphate kinase, have been investigated both experimentally and computationally [9, 23]. Two studies of polyphosphate kinase experimentally support polyphosphate kinase as a promising drug target [23, 24] while a study of cytidylate kinase did not publically deposit the predicted model structure and no inhibitors were been identified [9]. Supported by our findings, cytidylate and polyphosphate kinase would thus appear to be relevant *M. tuberculosis* drug targets. The three remaining proteins from our study, Rv2195, Rv1456c and Rv2421c have, to our knowledge, not previously been proposed as drug targets.

### Pathway and drug target validation

The nine genes reported in this study form part of three metabolic pathways: pyrimidine, oxidative phosphorylation, nicotinate and nicotinamide metabolism. Of these, Rv1622c and Rv1456c constitute a two component system unique to bacteria, fungi and plants. Drugs inhibiting these targets would therefore be specific to bacteria with minimal human toxicity. The target Rv1712 is central to the phosphorylation of ATP to nucleoside diphosphates in the pyrimidine pathway [25]. The known TB drug target Rv0667 forms part of the purine and pyrimidine pathway and mutations in its gene *rpoB* lead to RIF resistance. With Rv1712 sharing this pathway it could be an attractive alternative target to inhibit the pathway.

Five candidates, Rv2984, Rv2194, Rv1311, Rv1305 and Rv2195, map to the oxidative phosphorylation pathway. The target Rv1854c (gene *ndh*) in this pathway is the target for INH and several mutations in this gene account for INH resistant cases [10, 26]. Inhibiting any of the five proposed targets could disrupt the pathway and eliminate *M. tuberculosis* by reducing its limited ATP availability during dormancy [14].

Rv2421c transfers phosphorous groups in nicotinate/nicotinamide salvage and *de novo* synthesis. Rv2043c of this pathway is the target of the highly effective drug PZA that kills persistent bacilli in the initial phase of TB therapy [27]. Mutations in the encoding gene *pncA* confer resistance to PZA [28]. Successful inhibition of Rv2421c could thus help to eradicate slowly growing persistent bacilli in TB infection.

Although the three selected pathways are also found in humans, the proposed targets are unique to bacteria and unrelated to human proteins meaning that possible drugs are unlikely to affect human metabolism. The similar absence of murine homologs could further allow for mouse infection studies. The nine targets are conserved in several mycobacteria pathogenic to humans such as *M. leprae*, *abscessus*, and *ulcerans*, implying that broad spectrum inhibitors are possible. As the targets are also conserved in *H. pylori, N. meningitidis* and *E. coli*, new TB drugs could be used to target these pathogens. The conservation of the targets underscores their essentiality and could imply some innate resistance to mutation. The proteins Rv2194, Rv2195 and Rv1456c lack close homologs in several human gut bacteria meaning that their inhibition by drugs is unlikely to disrupt the host microbiome. Although Rv1712, Rv2984, Rv1311, Rv1305, Rv1622c and Rv2421c share remote homologs in several gut bacteria, their investigation is warranted as the target for the TB drug Rifampicin, DNA-directed RNA polymerase, is highly conserved across all species. A mere five exchanged residues between eukaryotic and bacterial RNA polymerase gives rise to the selectivity of RIF [29]. While Rv1305 satisfied all our prioritization criteria, its conservation in humans demote it in our list of putative targets. Subtle differences between bacterial and human versions could nevertheless reinstate it Rv1305 as a potential target. Finally, Rv1712 was further analysed because our results and experimental findings indicate it to be essential for *M. tuberculosis* [13].

### Molecular dynamics

The simulated molecular dynamics of Rv1712 in complex with C5P indicated 30 ns to be sufficient for reaching a stability plateau. This is in contrast to the [9] study which found 3 ns to be sufficient for equilibrating the structure. However, the RMSF fluctuation of all C-alpha residues for our model was similar to that of the [9] study suggesting that the presence of ligand C5P introduces major stability of the LID domain (residues 155–168) of the protein preventing ATP hydrolysis. Additionally, the radius of gyration for the molecule became constant after 5000 ps suggesting that the Rv1712-C5P complex has a stable surface structure suitable for virtual screening and drug design.

### Virtual screening and interaction analysis

Computational methods have proven useful in the past to identify novel drugs to treat resistant strains of *Mycobacterium tuberculosis* [30]. Using similar approaches we have successfully identified ten compounds with higher binding affinity values compared to C5P docked to the equilibrated model of Rv1712. Analysis of these ten compounds showed a larger number of interactions namely hydrogen bonds and salt bridges compared to ligand C5P suggesting a stronger affinity for Rv1712. Further analysis revealed interaction of compounds with highly conserved key residues (Thr32, Gly33, Tyr36, Arg37, Asp129 and Arg185) important for catalytic activity of Rv1712 as identified in a study by [31] using the homologous template 2H92. Interestingly our compounds also interact with Lys14 which is part of the P-loop forming the large anion hole which usually binds the sulphate ion thereby preventing nucleophillic attack by the phosphate of ATP. This implies a role for these compounds as competitive inhibitors of Rv1712. Although ligand C5P makes only one hydrogen bond with residue Gln113 in our predicted model compared to the [9] study which makes ten hydrogen bonds, this is only due to a lower hydrogen bond cut-off value (>2.4 Å). Nine compounds share the overall orientation of C5P and its binding site. The evidence provided advocates the need for additional *in silico* studies to validate these compounds as inhibitors of Rv1712. We propose that the compounds should be tested experimentally to establish their non-toxicity to human cell lines and their inhibition of *M. tuberculosis* growth.

## Conclusions

In this study, known drug resistance genes were mapped to three KEGG metabolic pathways. All proteins from these three metabolic pathways were subjected to a filtering criteria such as essentiality, known function, absence of human homologs and conservation within *Mycobacteria.* This resulted in the identification of nine putative drug target candidates of which three are novel (Rv2195, Rv2421c and Rv1456c) The integrated approach successfully implemented in this study can be extended to other pathogenic organisms for which drug resistance data is available. Furthermore, ten compounds with higher affinity than substrate C5P was identified using molecular docking and these compounds warrants further investigation to assess their potential to inhibit *M. tuberculosis* growth.

## Methods

### Identification of potential M. tuberculosis targets

A comparison of the genomes of three KwaZulu-Natal TB strains, KZN 4207, KZN 1435 and KZN 605, identified ten mutations in eight genes that account for drug resistance and another 26 coding and non-coding polymorphisms in 20 further genes for differences between MDR and XDR strains [3]. We investigated the latter set of 20 proteins with respect to their metabolic pathways. Most (75 %) could not be assigned to any pathway and 40 % are annotated as hypothetical proteins. Only two genes map to protein transport and transcription. Ten additional genes associated with first and second-line TB drug resistance were identified from the literature. This study focuses on 18 essential genes associated with drug resistance [10].

### Metabolic pathway analysis and target prioritization

The 18 known drug resistance genes identifiers namely the Rv numbers were used to query the KEGG *M. tuberculosis H37Rv* strain database [11]. The Rv numbers were used as input to search the KEGG database which returned metabolic pathway diagrams showing the location of the specific target. We then extracted all the gene products by Rv number which provided us an opportunity to investigate other genes (excluding known genes) as drug candidates. Six of the 18 drug resistance genes have not been annotated with any pathway data and were assigned to what is called “pathway holes”. We therefore focused on targets that map to pathways containing first-line drug resistance genes as these pathways have been extensively characterized [32]. Three metabolic pathways were selected because of experimental evidence for gene products within these pathways associated with importance for bacterial growth during active and latent states and their potential for developing antimicrobial agents for other genes within these pathways [12–15]. These three pathways comprise 105 genes of which 14 are known TB genes that were excluded from our search for drug targets. Thirty-eight of the remaining 91 *H37rV* genes were not currently under investigation by the TB Structural Genome Consortium (TBSGC) as reported on the TBSGC web portal. Only one target currently being investigated by the TBSGC was included in our candidate list because no structure has yet been elucidated for this protein.

The TubercuList World Wide Web Server [33] was used to query the combined 39 genes based on their Rv number to find relevant information regarding essentiality of the gene, if its function is known (meaning it has a biological role within an reaction step of the pathway) and if a 3D structure is known. TubercuList provided valuable experimental evidence for growth based on transposon site hybridisation assays [13, 34] and other mutagenic studies done on all *M. tuberculosis* genes confirming their essentiality or non-essentiality. Seventeen of the 39 potential drug targets can be classified as essential genes with known biological function and were used for BLASTp searches against the *Homo sapiens* database at NCBI (build GRCh37/hg19) [35]. Among these 17 proteins, nine showed no similarity to human proteins and were chosen as potential drug targets, eliminating possible human host protein-drug interactions (expectation score <0.0005). The amino acid sequence of each of the nine selected drug candidates were used to perform a BLASTp search against 24 *Mycobacterium* species to determine inter-species sequence conservation (Additional file 4: Table S4). A high degree of sequence conservation suggests that mutations in these proteins are not tolerated thereby prohibiting the spontaneous occurrence of drug resistance. The *M. tuberculosis* targets that were retained were investigated for their possible role in *M. tuberculosis* survival during latency conditions. Genes that are essential for dormancy survival are up-regulated during latent conditions of *M. tuberculosis* and should be targeted in an attempt to combat TB persistence. The *M. tuberculosis* protein accession numbers were used to query the Tuberculosis Database [36] to obtain gene expression data collated for each target. These include samples, conditions and the statistically significant measurements (*p*-value) for each experiment. Conditions most often associated with persistence have been reported as hypoxia, starvation and change in PH [37]. Additionally, the lack of close homologs to human gut flora would facilitate the design of *M. tuberculosis* specific drugs and inform in vivo mouse infection studies. To this end, protein BLAST searches were carried out against three species of human gut flora bacteria *Staphylococcus aereus* (taxid: 1280), *Enterococcus faecalis* (taxid: 1351) and *Escherichia Coli* (taxid: 562) and mouse proteins (*Mus musculus*).

### Homology modeling

Homology models were constructed for *M. tuberculosis* cytidylate kinase, Rv1712, using Modeller version 9.7 [38]. Homologous template structures were identified by amino-acid sequence alignment against those of the Protein Data Bank (PDB) [39] using the profile.build module of Modeller. Multiple sequence alignments were obtained using the salign routine. The manually optimized alignment was used by Modeller model-mult-hetero module to construct the models based on satisfaction of spatial restraints. Structural templates for Rv1712 were cytidine monophosphate kinase (CMPK) from *Streptococcus pneumoniae* (1Q3T), cytidylate kinase from *Staphylococcus aureus* in complex with cytidine-5-monophosphate (2H92), and CMPK from *E. coli* both without (1CKE) and with C5P (1KDO). We randomly generated fifty models for Rv1712 using Modeller in the presence of ligand C5P.

### Model evaluation

The lowest Dope score model (LDSM) was selected for qualitative analysis as it presumably is closest to the native protein structure [40]. The discrete optimized protein energy (DOPE) score energy profiles were calculated using evaluate_model.py for the LDSM and the templates and compared graphically using Gnuplot (version 4.2) to locate regions of high energy [41]. The quality of the LDSM was assessed using the normalised DOPE Z-scores (average score of all the heavy C-alpha atom pairs) calculated using assess_normalised_dope.py with DOPE Z-scores below −1 indicating acceptable models. The reliability of regions involved in substrate binding were assessed using Prosaii [42] which indicates overall model quality (Z-score) and measures the deviation of the total energy of the structure with respect to an energy distribution derived from random conformations [43, 44] while the stereochemical quality was checked using Procheck [17]. The quality of the LDSM with respect to non-bonded atom-atom interactions was assed using Errat [18]. Finally, the structural similarity of model and the four templates were assessed using root mean square deviations (rmsd) as calculated in PyMol [45] by aligning all atoms to one another.

### Molecular dynamics

The LDSM was energy minimized by 1000 steps of steepest descent and 5000 steps of conjugated gradients using Gromacs. The energy minimized structure was then subjected to 30 ns of molecular dynamics simulation to determine stability of the structure in complex with ligand C5P also in Gromacs [46]. The simulation was repeated using random numbers for generating velocities to validate the reproducibility of the results verifying that all observables including the trajectory converge to reach their equilibrium values. The total and potential energy terms were calculated using Gromacs tool g_energy. Also, the backbone atoms RMSD and C-alpha atoms RMSF values was calculated using Gromacs utilities g_rms and g_rmsf for the equilibrated structure over the whole trajectory. RMSF is a measure of deviation between the position of a particle and some reference position averaged over time. The radius of gyration was calculated for all backbone atoms using Gromacs utility g_gyrate.

### Parameter optimisation

The ligand C5P was docked to the equilibrated model of Rv1712’s active site; Asp31, Tyr36, Arg37, Arg106, Arg128, Asp129, Arg178, Asp182 and Arg185 using AutoDock-vina. Vina implements a sophisticated gradient optimisation method in its local optimisation step and an pairwise empirical scoring function to find the global energy minimum conformation of a ligand. Vina was selected for rigid body docking because it has been validated for its accuracy in reproducing experimental conformations. The top ten binding modes produced by the docking run of the ligand C5P were compared to the most stable ligand trajectory by visualizing the output file and input structure in Pymol. This was done to determine if AutoDock-vina correctly predicted the top binding conformation of the ligand as the most energy favourable and if it can replicate the most stable ligand trajectory *in silico*.

### Molecular docking

#### Docking C5P to the equilibrated structure

The equilibrated protein structure Rv1712.gro was converted to pdb format by editconf and sodium atoms, solvent and C5P molecules were deleted because no optimal force field has been defined for these molecules. The final Rv1712 model and C5P were converted to a set of united-atom aliphatic carbons (force field where all hydrogens on aliphatic carbons are united with carbons), aromatic carbons, polar hydrogens, hydrogen bonding nitrogens and oxygens with charges called pdbqt using AutoDock Tools [47] to correct for errors such as missing atoms, added H_2_O, more than one molecule chain breaks instead of one, and alternate locations of rotamers for side chains. The receptor’s binding site was kept fixed while seven bonds within the ligand was treated as rotatable. Binding energies were calculated and the docked ligand pose was visualised using Pymol. The binding energies reported represent the sum of the intermolecular energy, total internal energy and torsional free energy minus the energy of the unbound system.

### Compound screening and interaction analysis

The ZINC database was searched using the simple molecular input line entry (SMILE) format of ligand C5P implementing an axonpath fingerprint search to identify structurally similar compounds. After screening, 48 compounds were identified at 95 % structural similarity and downloaded in sdf format from the ZINC database. The compounds were converted to pdbqt format using Open Babel [48] in preparation for docking simulation. The processed compounds were docked to the overall simulated protein model of Rv1712 using AutoDock Vina software [49]. Rigid body docking was performed to obtain several possible conformations and orientations for the compounds docked at the receptor’s active site. The Grid box dimensions of previously parameter optimised dockings were implemented. Docking energies calculated during the run were extracted and ranked using an python script called vina_screen_get_top.py with lowest energy values at the top. The top ten lowest energy binding modes for each compound were visually inspected in PyMol and were further analysed using PoseView and BINANA programs [50, 51]. PoseView determines four types of interactions namely; i) hydrogen bonds, ii) hydrophobic, iii) metal interactions and iv) π interactions, while BINANA was used to calculate all the afore mentioned interactions as well as salt bridges because this functionality was not present in PoseView.

### Availability of supporting data

The scripts, simulation data and docking data for ten compounds used in these analyses are available on the South African National Bioinformatics Institute permanent data archive (ftp://ftp.sanbi.ac.za/tb_targets/Rv1712). All other supporting data are included as additional files.

## Additional files

Additional file 1: Table S1. KEGG pathways for genes known to be associated with 1st and 2nd line drug resistance. The 10 mutations (eight genes) indicated in bold were verified by the BROAD institute, while the remainder were identified from literature. Abbreviations used: A-Adenine, Ala-Alanine, Arg-Arginine, Asn-Glutamine, Asp-Aspartate, Gly-Glycine, His-Histidine, indel-insertion deletion, Ileu-Isoleucine, Leu-Leucine, Met-Methionine, N.A-Not available, Pro-Proline, Ser-Serine, T-Thymidine, Thr-Threonine, Tyr-Tyrosine, Val-Valine. (PDF 143 kb) Additional file 2: Table S2. Criteria used to filter high priority *M.tuberculosis* drug targets. The genes highlighted in bold satisfied all the selection criteria. The hyphen (−) indicates exclusion from further analysis. Abbreviations used: NUI- Not under investigation, PDB- Protein Data Bank, TBSGC- TB Structural Genome Consortium. References 12-Sassetti et al., 2003; 34-Lamichhane et al., 2003. Data can be viewed in Microsoft excel. (XLS 12 kb) Additional file 3: Table S3. The 24 sequenced *Mycobacterium* strains. Data can be viewed in Microsoft excel. (XLS 34 kb) Additional file 4: Table S4. Blast hits obtained for Rv1712, Rv2984, Rv2194, Rv1311, Rv1305, Rv2195, Rv1622c, Rv1456c and Rv2421c against three host intestinal bacteria. Bacterial species highlighted in bold showed homology to the query gene. Data can be viewed in Microsoft excel. (XLS 13 kb) Additional file 5: Figure S1. KEGG metabolic pathway map for Nucleotide metabolism (pyrimidine metabolism) in *M. tuberculosis H37rV* strain. Rv1712 or *cmk* selected for investigation is shown in red highlighted boxes and involved in step 2.7.4.14 of this specific pathway. Known drug resistance gene Rv0667 or *rpoB* is shown in blue highlighted box and involved in step 2.7.7.6 of this pathway. *M. tuberculosis* specific genes are coloured in green. (PDF 111 kb) Additional file 6: Figure S2. KEGG metabolic pathway map for Oxidative phosphorylation in *M. tuberculosis H37rV* strain. Rv2984 or *ppk* selected for investigation is shown in blue highlighted box and involved in step 2.7.4.1 of this specific pathway. Rv2194 or *qcrC* selected for investigation is shown in the red highlighted box. Both Rv1305 (*atpE*) and Rv1311 (*atpC*) selected for investigation are shown in brown highlighted box and involved in step 3.6.3.14 of this specific pathway. Rv2195 or *qcrA* selected for investigation is shown in the yellow highlighted box involved in cytochrome C reductase. Rv1456c or COX15 selected for investigation is shown in the magenta highlighted box involved in cytochrome C oxidase. Rv1622c or *CydB* selected for investigation is shown in the light blue highlighted box involved in cytochrome C oxidase. Known drug resistance gene Rv1854c or *ndh* is shown in orange highlighted box and involved in step 1.6.99.3 of this pathway. *M. tuberculosis* specific genes are coloured in green. (PDF 157 kb) Additional file 7: Figure S3. KEGG metabolic pathway map for Nicotinate and Nicotinamide metabolism in *M. tuberculosis H37rV* strain. Rv2421c or *nadD* selected for investigation is shown in red highlighted box and involved in step 2.7.7.18 of this specific pathway. Known drug resistance gene Rv2043c or *pncA* is shown in blue highlighted box and involved in step 3.5.1.19 of this pathway. *M. tuberculosis* specific genes are coloured in green. (PDF 106 kb) Additional file 8: Table S5. KEGG pathway descriptors for *M. tuberculosis* genes retained after prioritization. Data can be viewed in Microsoft excel. (XLS 7 kb) Additional file 9: Figure S4. DOPE score energy profiles graph of the structural model for Rv1712 (red) and templates 1Q3T (green), 1CKE (dark blue), 1KDO (light blue) and 2H92 (purple). Generated using Gnuplotv4.2 [41]. (PDF 68 kb) Additional file 10: Figure S5. RMSD of the backbone atoms of model Rv1712 (green) and substrate C5P (red) during the 30000 ps simulation. Generated using Gnuplotv4.2 [41]. (PDF 198 kb) Additional file 11: Figure S6. The variation in total (green) and potential energy (red) for the Rv1712-C5P complex during the 30000 ps simulation. Generated using Gnuplotv4.2 [41]. (PDF 69 kb) Additional file 12: Figure S7. RMS fluctuations of all Cα residues for Rv1712 over the 30000 ps simulation. (PDF 55 kb) Additional file 13: Figure S8. Radius of gyration of all bacbone atoms for Rv1712 over the 30000 ps simulation. (PDF 29 kb) Additional file 14: Figure S9. Superimposition of lowest DOPE score models for the initial and newly generated structures. The blue model represents the initial model without 3R20 used as a template while the red model consist of 3R20 used as a template for model construction. Ligands SO4 and CMP are shown as sticks. RMSD = 0.387 Å. (PDF 33 kb) Additional file 15: Table S6. Docking scores and number of interactions for the top ten compounds to Rv1712. The residues highlighted in bold are conserved catalytic residues while ligand C5P is highlighted in red. The numbers inside the () indicate the number of interactions formed between residue and the compound atom. Data can be viewed in Microsoft excel. (XLS 8 kb)
