# Supplementary material for: Resistance related metabolic pathways for drug target identification in Mycobacterium tuberculosis
Source: BMC Bioinformatics. 2016 Feb 8;17:75. doi: 10.1186/s12859-016-0898-8 (PMC4745158; doi:10.1186/s12859-016-0898-8)
Supplement: Additional file 1: Table S1. — KEGG pathways for genes known to be associated with 1st and 2nd line drug resistance. The 10 mutations (eight genes) indicated in bold were verified by the BROAD institute, while the remainder were identified from literature. Abbreviations used: A-Adenine, Ala-Alanine, Arg-Arginine, Asn-Glutamine, Asp-Aspartate, Gly-Glycine, His-Histidine, indel-insertion deletion, Ileu-Isoleucine, Leu-Leucine, Met-Methionine, N.A-Not available, Pro-Proline, Ser-Serine, T-Thymidine, Thr-Threonine, Tyr-Tyrosine, Val-Valine. (PDF 143 kb) [file 12859_2016_898_MOESM1_ESM.pdf]

Drug	Gene(s)	Mutation	Pathway
INH (Isoniazid) 1st	Rv1484 (inhA)	**-8 T/A** and Ser94Ala	mycolic acid biosynthesis
	Rv1908c (katG)	**Ser315Thr**	tryptophan metabolism and methane metabolism
	Rv3795 (embB)	Met306Ileu	arabinosyl transferase
	Rv2428 (ahpC)	Ser315Thr	glutathione metabolism
	Rv2245 (kasA)	Asp66Asn	N.A
	Rv1854c (ndh)	Thr110Ala and Arg268His	oxidative phosphorylation
PZA (Pyrazinamide) 1st	Rv2043c (pncA)	**Ala132Gly**, Thr114Met	nicotinate and nicotinamide metabolism
RIF (Rifampicin) 1st	Rv0667 (rpoB)	**Asp435Gly, Leu452Pro, Ile1106Thr,** Ser531Leu or His526Tyr	purine metabolism, pyrimidine metabolism, RNA polymerase
	Rv3795 (embB)	**Met306Val**	arabinosyl transferase
EMB (Ethambutol) 1st	Rv3795 (embB)	Met306Ileu	arabinosyl transferase
	Rv3793 (embC)	Met306Val	arabinosyl transferase
	Rv3794 (embA)	Met306Ileu	arabinosyl transferase
	Rv3266c (rmlD)	N.A	nucleotide sugars metabolism, streptomycin biosynthesis, polyketide sugar unit biosynthesis
STR (Streptomycin) 1st	Rv3919c (gidB)	**Indel**	N.A
ETH (Ethionamide) 2nd	Rv1483 (mapA, fabG1)	Ser94Ala	mycolic acid biosynthesis
	Rv3854c (ethA, ethR)	N.A	aminobenzoate degradation
FLQ (flouroquinolones) 2nd	Rv0005 (gyrB)	N.A	N.A
	Rv0006 (gyrA)	**Ala90Val**, Asp94Gly	N.A
AMI (Aminoglycosides) 2nd	MTB000019 (rrs)	**Ala1401Gly**	N.A
	Rv1694 (tlyA)	N.A	N.A

The 10 mutations (eight genes) indicated in bold were verified by the BROAD institute, while the remainder were identified from literature. Abbreviations used: A-Adenine, Ala-Alanine, Arg-Arginine, Asn-Glutamine, Asp-Aspartate, Gly-Glycine, His-Histidine, indel-insertion deletion, Ileu-Isoleucine, Leu-Leucine, Met-Methionine, N.A-Not available, Pro-Proline, Ser-Serine, T-Thymidine, Thr-Threonine, Tyr-Tyrosine, Val-Valine.
