# Supplementary material for: Resistance related metabolic pathways for drug target identification in Mycobacterium tuberculosis
Source: BMC Bioinformatics. 2016 Feb 8;17:75. doi: 10.1186/s12859-016-0898-8 (PMC4745158; doi:10.1186/s12859-016-0898-8)
Supplement: Additional file 6: Figure S2. — KEGG metabolic pathway map for Oxidative phosphorylation in M. tuberculosis H37rV strain. Rv2984 or ppk selected for investigation is shown in blue highlighted box and involved in step 2.7.4.1 of this specific pathway. Rv2194 or qcrC selected for investigation is shown in the red highlighted box. Both Rv1305 (atpE) and Rv1311 (atpC) selected for investigation are shown in brown highlighted box and involved in step 3.6.3.14 of this specific pathway. Rv2195 or qcrA selected for investigation is shown in the yellow highlighted box involved in cytochrome C reductase. Rv1456c or COX15 selected for investigation is shown in the magenta highlighted box involved in cytochrome C oxidase. Rv1622c or CydB selected for investigation is shown in the light blue highlighted box involved in cytochrome C oxidase. Known drug resistance gene Rv1854c or ndh is shown in orange highlighted box and involved in step 1.6.99.3 of this pathway. M. tuberculosis specific genes are coloured in green. (PDF 157 kb) [file 12859_2016_898_MOESM6_ESM.pdf]

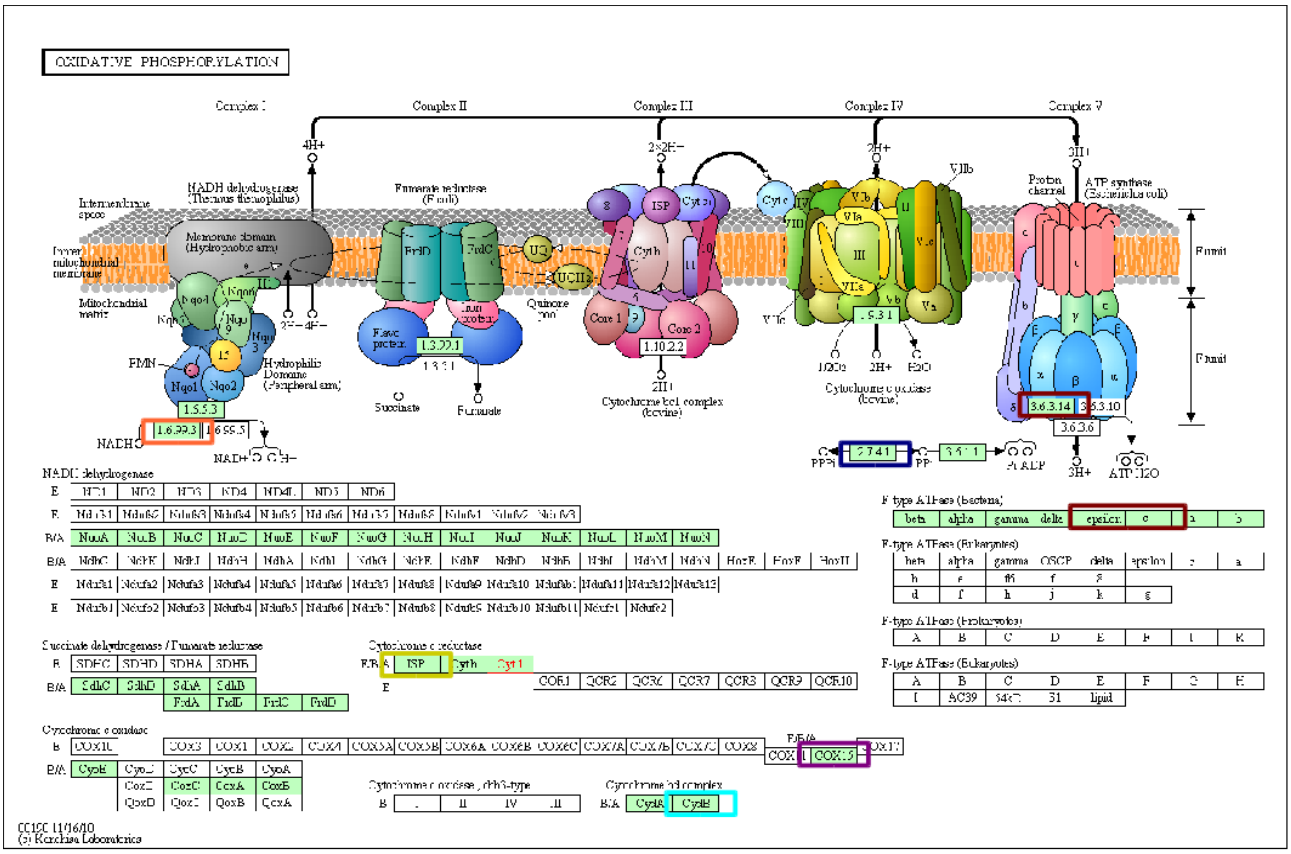

Figure S2: KEGG metabolic pathway map for Energy metabolism (oxidative phosphorylation) in *M. tuberculosis* H37rV strain.

Rv2984 or *ppk* drug target candidate is shown in blue highlighted box and involved in step 2.7.4.1 of this specific pathway. Rv2194 or *qcrC* drug target candidate is shown in the red highlighted box. Both drug target candidates Rv1305 (*atpE*) and Rv1311 (*atpC*) are shown in brown highlighted box and involved in step 3.6.3.14 of this specific pathway. The proposed drug target Rv2195 or *qcrA* is shown in the yellow highlighted box and involved in cytochrome C reductase. Drug candidates Rv1456c or COX15 is shown in the magenta highlighted box involved in cytochrome C oxidase and Rv1622c or *CydB* is shown in the light blue highlighted box involved in ubiquinol-cytochrome oxidase. Known drug resistance gene Rv1854c or *ndh* is shown in orange highlighted box and involved in step 1.6.99.3 of this pathway. *M. tuberculosis* specific genes are coloured in green.
