# Supplementary material for: Resistance related metabolic pathways for drug target identification in Mycobacterium tuberculosis
Source: BMC Bioinformatics. 2016 Feb 8;17:75. doi: 10.1186/s12859-016-0898-8 (PMC4745158; doi:10.1186/s12859-016-0898-8)
Supplement: Additional file 7: Figure S3. — KEGG metabolic pathway map for Nicotinate and Nicotinamide metabolism in M. tuberculosis H37rV strain. Rv2421c or nadD selected for investigation is shown in red highlighted box and involved in step 2.7.7.18 of this specific pathway. Known drug resistance gene Rv2043c or pncA is shown in blue highlighted box and involved in step 3.5.1.19 of this pathway. M. tuberculosis specific genes are coloured in green. (PDF 106 kb) [file 12859_2016_898_MOESM7_ESM.pdf]

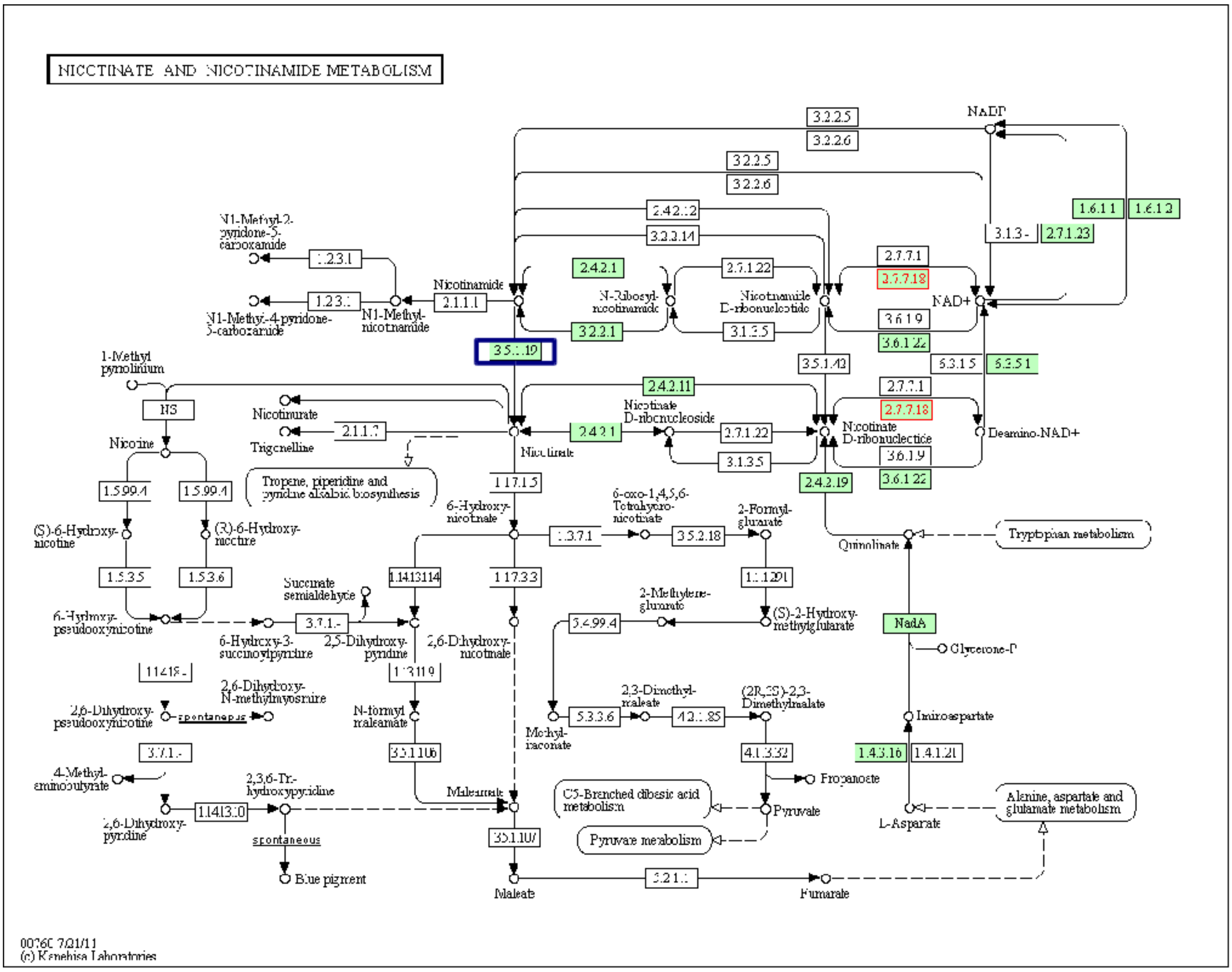

Figure S3: KEGG metabolic pathway map for Nicotinate and Nicotinamide metabolism in *M. tuberculosis* H37rV strain.

Rv2421c or *nadD* drug target candidate is shown in red highlighted box and involved in step 2.7.7.18 of this specific pathway. Known drug resistance gene Rv2043c or *pncA* is shown in blue highlighted box and involved in step 3.5.1.19 of this pathway. *M. tuberculosis* specific genes are coloured in green.
