# Supplementary material for: Resistance related metabolic pathways for drug target identification in Mycobacterium tuberculosis
Source: BMC Bioinformatics. 2016 Feb 8;17:75. doi: 10.1186/s12859-016-0898-8 (PMC4745158; doi:10.1186/s12859-016-0898-8)
Supplement: Additional file 9: Figure S4. — DOPE score energy profiles graph of the structural model for Rv1712 (red) and templates 1Q3T (green), 1CKE (dark blue), 1KDO (light blue) and 2H92 (purple). Generated using Gnuplotv4.2 [41]. (PDF 68 kb) [file 12859_2016_898_MOESM9_ESM.pdf]

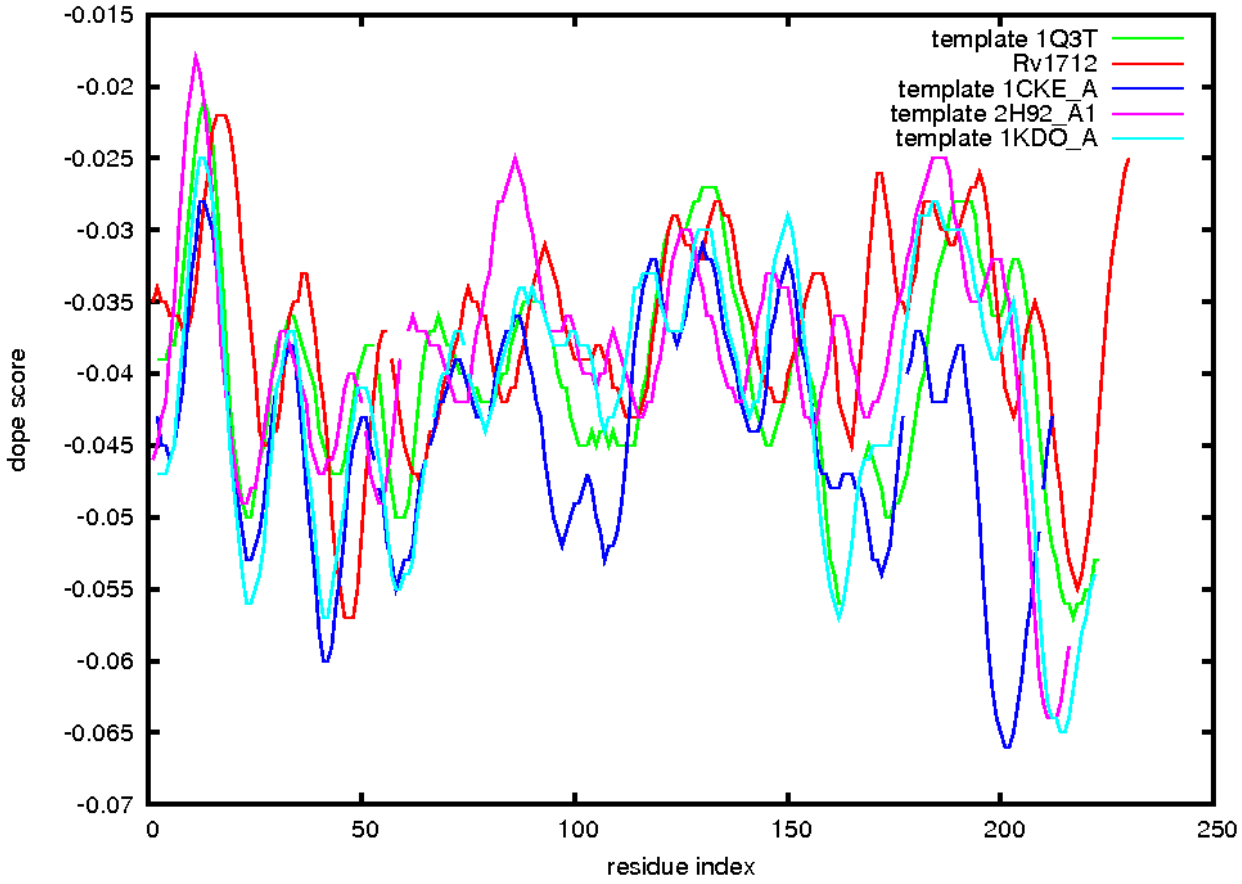

Figure S4 - DOPE score energy profiles graph of the structural model for Rv1712 (red) and templates 1Q3T (green), 1CKE (dark blue), 1KDO (light blue) and 2H92 (purple).
Generated using Gnuplotv4.2 [41].
