# Supplementary material for: Resistance related metabolic pathways for drug target identification in Mycobacterium tuberculosis
Source: BMC Bioinformatics. 2016 Feb 8;17:75. doi: 10.1186/s12859-016-0898-8 (PMC4745158; doi:10.1186/s12859-016-0898-8)
Supplement: Additional file 12: Figure S7. — RMS fluctuations of all Cα residues for Rv1712 over the 30000 ps simulation. (PDF 55 kb) [file 12859_2016_898_MOESM12_ESM.pdf]

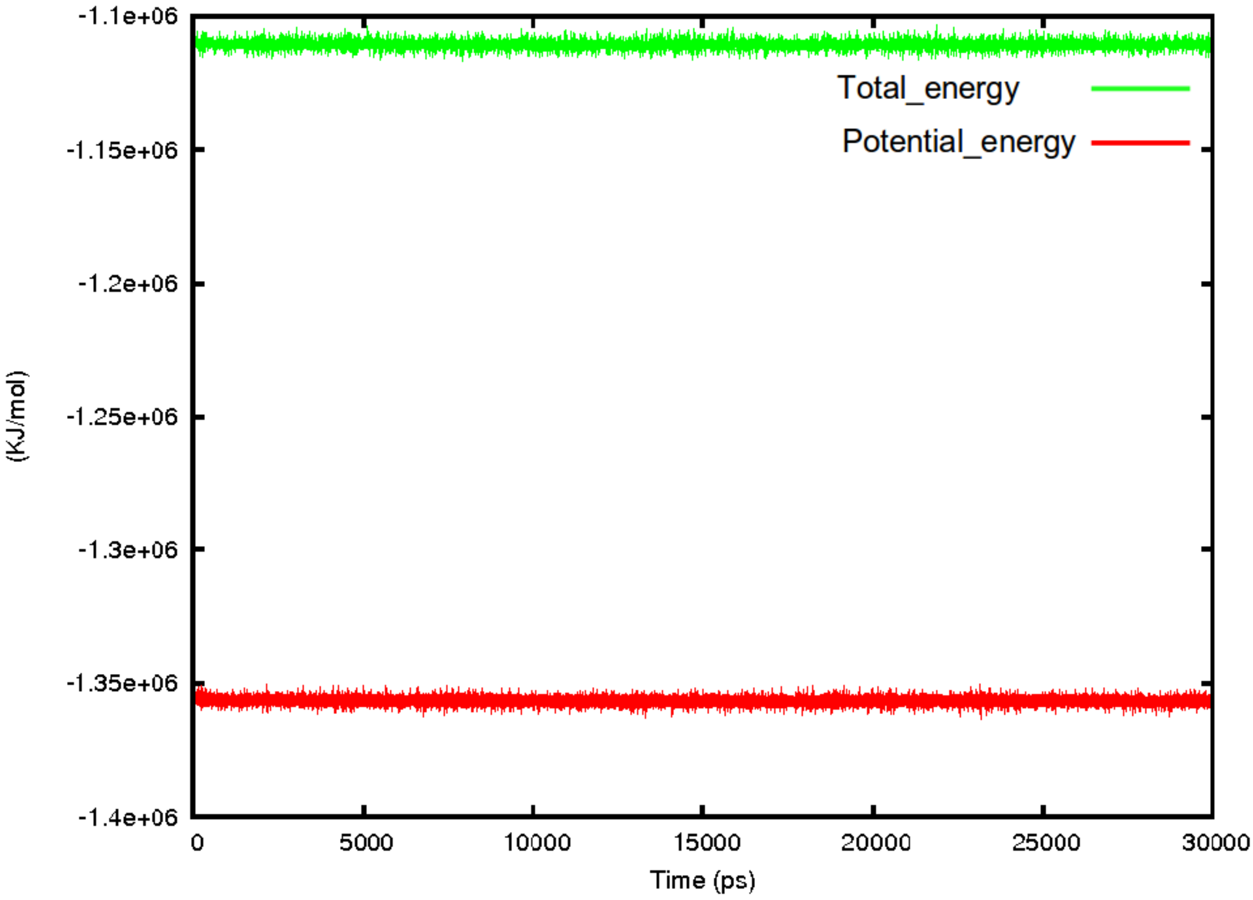

Figure S7 – The variation in total (green) and potential energy (red) for the Rv1712-C5P complex during the 30000ps simulation.
Generated using Gnuplotv4.2 [41].
