# Supplementary figures and images for: Resistance related metabolic pathways for drug target identification in Mycobacterium tuberculosis
Source: BMC Bioinformatics. 2016 Feb 8;17:75. doi: 10.1186/s12859-016-0898-8 (PMC4745158; doi:10.1186/s12859-016-0898-8)

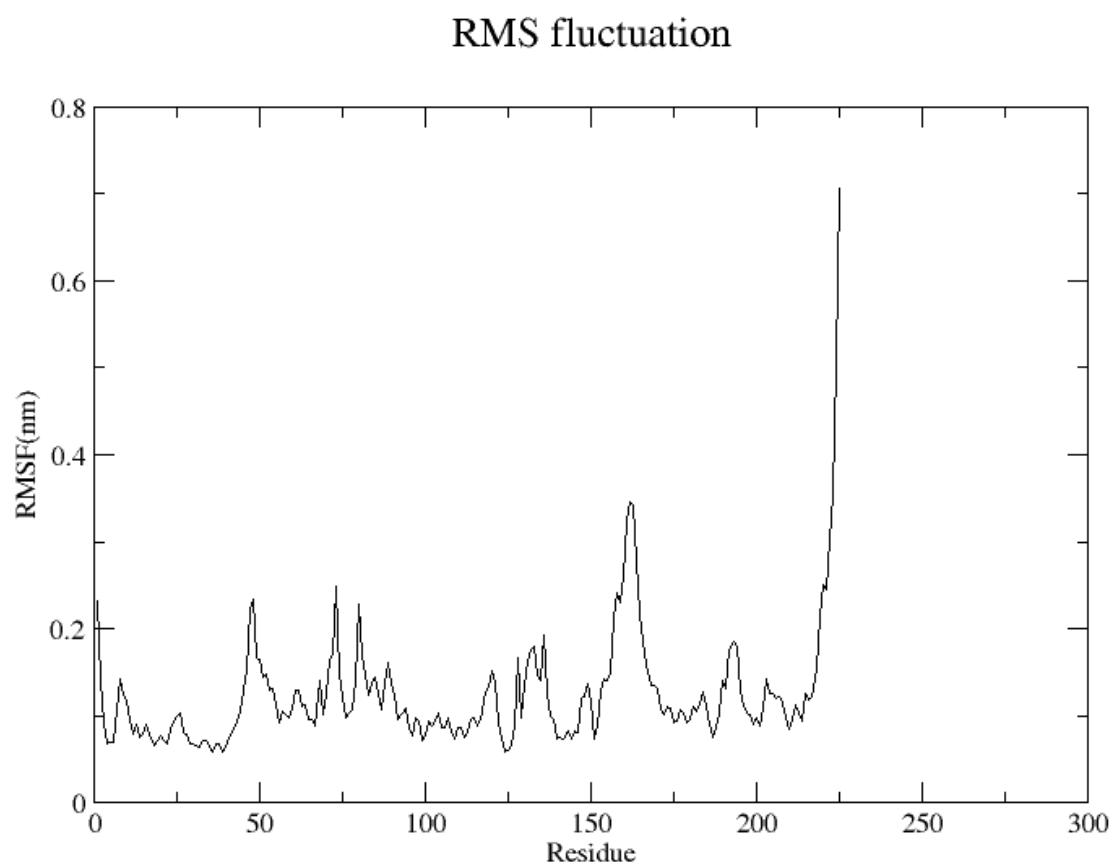

**Figure S8 – RMS fluctuations of all Cα residues for the Rv1712 over the 30000ps simulation.**

Supplement: Additional file 13: Figure S8. — Radius of gyration of all bacbone atoms for Rv1712 over the 30000 ps simulation. (PDF 29 kb) [file 12859_2016_898_MOESM13_ESM.pdf]
