# Supplementary material for: Resistance related metabolic pathways for drug target identification in Mycobacterium tuberculosis
Source: BMC Bioinformatics. 2016 Feb 8;17:75. doi: 10.1186/s12859-016-0898-8 (PMC4745158; doi:10.1186/s12859-016-0898-8)
Supplement: Additional file 14: Figure S9. — Superimposition of lowest DOPE score models for the initial and newly generated structures. The blue model represents the initial model without 3R20 used as a template while the red model consist of 3R20 used as a template for model construction. Ligands SO4 and CMP are shown as sticks. RMSD = 0.387 Å. (PDF 33 kb) [file 12859_2016_898_MOESM14_ESM.pdf]

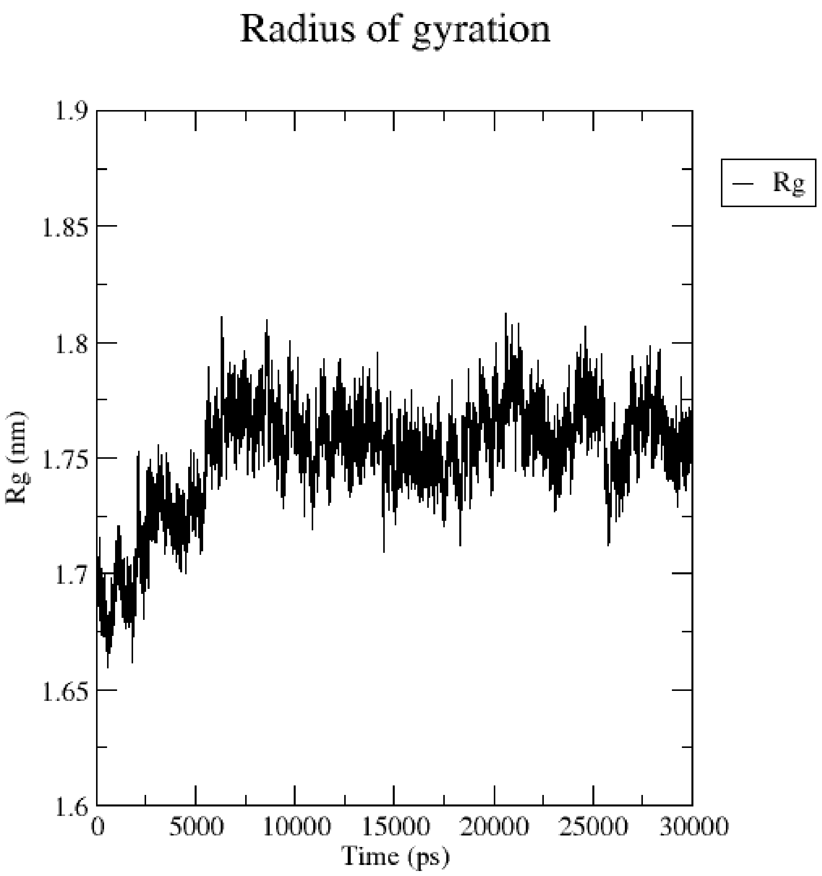

Figure S9 – Radius of gyration of all backbone atoms for Rv1712 over the 30000ps simulation.
