# Supplementary material for: Resistance related metabolic pathways for drug target identification in Mycobacterium tuberculosis
Source: BMC Bioinformatics. 2016 Feb 8;17:75. doi: 10.1186/s12859-016-0898-8 (PMC4745158; doi:10.1186/s12859-016-0898-8)
Supplement: Additional file 5: Figure S1. — KEGG metabolic pathway map for Nucleotide metabolism (pyrimidine metabolism) in M. tuberculosis H37rV strain. Rv1712 or cmk selected for investigation is shown in red highlighted boxes and involved in step 2.7.4.14 of this specific pathway. Known drug resistance gene Rv0667 or rpoB is shown in blue highlighted box and involved in step 2.7.7.6 of this pathway. M. tuberculosis specific genes are coloured in green. (PDF 111 kb) [file 12859_2016_898_MOESM5_ESM.pdf]

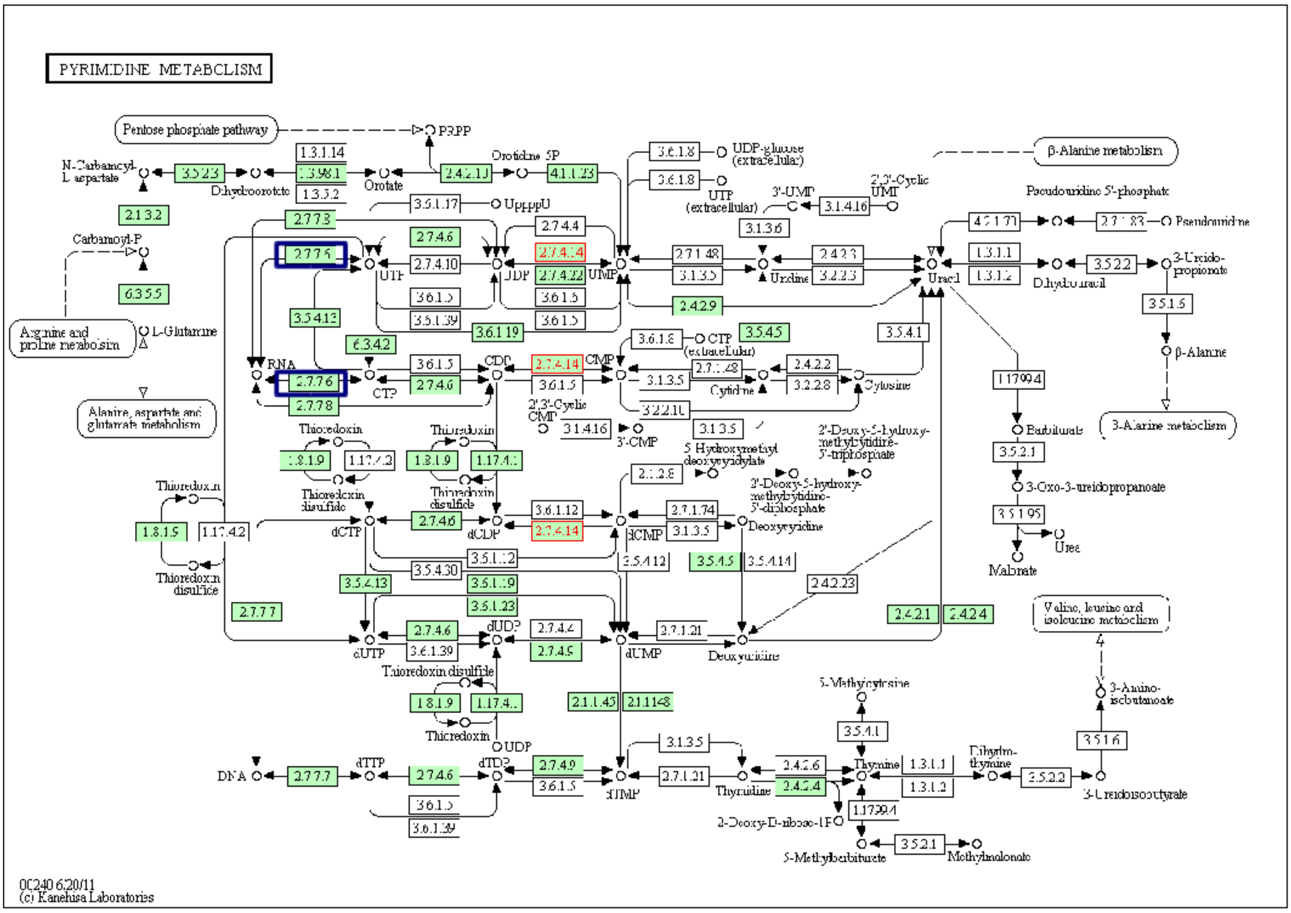

Figure S1: KEGG metabolic pathway map for Nucleotide metabolism (pyrimidine metabolism) in *M. tuberculosis* H37rV strain.

Rv1712 or *cmk* drug target candidate is shown in red highlighted boxes and involved in step 2.7.4.14 of this specific pathway. Known drug resistance gene Rv0667 or *rpoB* is shown in blue highlighted box and involved in step 2.7.7.6 of this pathway. *M. tuberculosis* specific genes are coloured in green.
